# Inflammatory and proapoptotic effects of inhaling gasoline fumes on the lung and ameliorative effects of fenugreek seeds

**DOI:** 10.1038/s41598-022-18607-9

**Published:** 2022-08-24

**Authors:** Abeer E. Abdrabouh

**Affiliations:** Zoology Department, Faculty of Science, Mansoura University, Mansoura, Egypt

**Keywords:** Zoology, Environmental sciences, Biomarkers

## Abstract

Impacts of inhaling gasoline fumes on the lungs of adult male rats and the alleviating role of fenugreek seeds were evaluated. Twenty-four rats were divided into four groups, unexposed control and fenugreek groups, gasoline exposed groups for 6 h/6 day/week for 10 weeks with and without supplementation of fenugreek seed powder in food (5% w/w). Rats exposed to gasoline fumes showed significant elevation in lung tumor necrosis factor-α, as an inflammatory marker, and the proapoptotic marker Bax with a reduction in the antiapoptotic marker Bcl2. Moreover, remarkable elevations in transforming growth factor-β1, collagen and hydroxyproline were observed as fibrotic markers. Lung oxidative stress markers (hydrogen peroxides, malondialdehyde, and protein carbonyl) increased significantly along with marked decrease in total antioxidant capacity, superoxide dismutase, and catalase levels. Additionally, marked decreases in white and red blood cell counts, hemoglobin content, platelet count, accompanied by elevated red cell distribution width percentage were observed, supporting the inflammatory status. Histopathological changes represented by hematoxylin&eosin, immunohistochemistry staining for Bax&Bcl2, and transmission electron microscopy supported the negative impacts of gasoline fumes compared to the control group. Fenugreek seeds supplementation with gasoline exposure showed pronounced alleviation of lung biochemical and histopathological changes compared to the gasoline-exposed group.

## Introduction

Environmental pollutants are a highly debated topic. One of the major occupational and environmental health concerns is exposure to gasoline fumes. Increasing concern has been directed to environmental safety and health hazards by providing unleaded RON 95 gasoline, but leaded RON 80 gasoline is still used in a broad category^1,2^. This type of gasoline contains noxious volatile organic compounds (VOCs), such as benzene, xylene, and toluene, that blend with harmful heavy metal additives, especially tetraethyllead compounds^3^. Benzene is known to have negative impacts on the hematopoietic system, and most occupational studies related this to the cytotoxic interactions of compounds, especially benzene with the bone marrow^2,4^. However, lead toxicity has a great public health concern due to its pervasiveness in the environment and wide toxic effects even at lower than considered^5^. Apoptosis is a normal physiological phenomenon in the body but may lead to pathological changes^6^. Exposure to lead compounds was reported to induce apoptosis in several organs, such as the liver and kidney^7^, brain cells^8^ and female ovaries^9^. Evidence suggests that in acute lung injury, an increase inflammatory marker TNF-α is closely related to induction of apoptosis in the alveolar epithelium^10^. Gasoline fume recovery systems are not commonly used in filling stations in most countries^4,11^. Thus, exposure to gasoline fumes was reported to be concerned with throat irritation, breathing difficulties, and a decrease in respiratory functions^12^. Moreover, gasoline fumes can easily reach the lung, diffuse along a wide surface area, and penetrate circulation^13^ and may lead to cancer^14^. In turn, the mixture of both VOCs and leaded compounds in gasoline may be a potent source of reactive oxygen species (ROS) that may stimulate the signaling cascades of the inflammatory process. The latter may act as a mediator of adverse health effects related to several pathogenic mechanisms including apoptosis and fibrosis^15^.

Since ancient times, plants with medicinal properties have been widely used to assist body functions and to treat different diseases^16^. Fenugreek (*Trigonella foenum-graecum*), family Leguminosae, is one of the most famous Mediterranean plants, originally seen in the Middle East and India and widely distributed throughout the world^17^. Its leaves and seeds have been used to prepare extracts for medical uses^18^. Seeds are rich in polyphenolic flavonoids, steroid saponins, and polysaccharides such as galactomannan^19^, in addition to vitamins (A, C, D, B_1_) and minerals (Ca, Fe, Zn)^20^. Moreover, Goyal et al.^21^ reported that seeds have cleansing action in purifying the blood and lymphatic system and detoxifying the body. Thus, fenugreek seeds have been identified to possess variable pharmacological effects including antioxidative^17^, antidiabetic^22^, anti-inflammatory, antifibrotic^16^, and antitumor^23^.

Therefore, the exact pathophysiological mechanism of gasoline exposure on the lungs is not fully understood, so this research mimicked occupational exposure to gasoline fumes to show negative impacts on the lungs of adult male rats. In addition, the present study speculated the role of fenugreek seeds in ameliorating the negative impacts of exposure to gasoline fumes.

## Materials and methods

### Experimental animals

Twenty-four healthy male Wistar albino rats (175–180 g) were obtained from the Egyptian Institute for Serological and Vaccine Production, Helwan, Egypt. In plastic cages, rats were acclimated for one week, taking food and water ad libitum under standard environmental conditions (12 h light and dark cycle, 23 ± 3 °C room temperature, and 40 ± 5% humidity). Used food components were previously mentioned at Abdrabouh^24^. The procedures used in this study were approved by the Animal Ethics Committee of Mansoura University, Egypt. I confirm that all methods were performed in accordance with the relevant guidelines and regulations.

### Preparation of fenugreek diet

Fenugreek seeds were purchased from commercial shop for spices in Mansoura City, that was grinded, then the powder was mixed with normal food (5% w/w). This was prepared weekly in pellet form, as mentioned by El-Wakf et al.^25^.

### Exposure to gasoline fumes

The Egyptian commercial leaded gasoline RON 80 was purchased from a filling station at Mansoura City. Rats were exposed to gasoline fumes through a stainless-steel exposure chamber with dimensions of (1.5 m × 0.9 m × 2.1 m), provided with two upper holes (10 cm diameter) and two side holes (5 cm diameter) in other sides, except the lower side. Two calibrated 1000 ml beakers, each containing 500 ml of leaded gasoline, were placed at the bottom of the chamber. Beakers were allowed one hour before the exposure to ensure chamber saturation with gasoline fumes, as reported by Uboh et al.^26^. Gasoline exposure was continued for 10 successive weeks, 6 h daily, and 6 days/week. The average volume of freely vaporized gasoline daily during the time of exposure was approximately 16 cm^3^.

### Experimental protocol

After the acclimation period, rats were classified into four groups (n = 6 per group): the control (CN) group, rats that received normal food and water, fenugreek (FN) group, rats supplemented with fenugreek seed powder mixed with normal food (5% w/w). Neither the CN nor FN groups were exposed to gasoline fumes or any source of pollution. In the gasoline (GS) group, rats were exposed to gasoline fumes as mentioned above, and in the (GS + FN) group, a fenugreek diet (5% w/w) was administrated during gasoline exposure.

This research was done in accordance with the ARRIVE guidelines and regulations for animal experiments.

### Blood and tissue sampling

Twenty-four hours after the last day of exposure, rats were sacrificed after anesthesia by intraperitoneal (I.P) injection of a mixture of ketamine (0.08 ml/g) and xylazine (0.008 ml/g) where each rat received (0.001 ml/g) from this mixture.

Blood samples were received on EDTA to estimate hematological parameters, including white blood cells (WBCs), red blood cells (RBCs) count, hemoglobin (Hb) content, hematocrit (HCT)%, mean corpuscular hemoglobin (MCH), mean corpuscular volume (MCV), red cell distribution width (RDW)% and total platelet (PLT) count, using a fully automatic hematological analyzer (Sysmex XE-2100, Japan) according to Dacie and Lewis^27^.

Next, some of the lung tissues of the different investigated groups were immediately removed, weighed, homogenized in cooled distilled water, and centrifuged at 855×*g* for 15 min. The supernatants were preserved at − 80 °C for biochemical analysis.

### Lung biochemical measurements

The following lung biochemical parameters were assessed according to protocols enclosed in enzyme-linked immunosorbent assay (ELISA) kits. The proinflammatory marker tumor necrosis factor-α (TNF-α) was estimated using the method provided by Alpco Diagnostics, USA, Catalog No. 45-TNFRT-E01.1. The proapoptotic marker (Bax) was detected using Cloud-Clone Corp, USA, Catalog No. SEB343Hu, and the antiapoptotic marker Bcl2 was detected by using MyBioSource, USA, Catalog No. MBS2512543. However, the fibrotic marker transforming growth factor-β1 (TGFβ1) was detected according to Cell Sciences, USA, Catalog No. 670.020.096. Collagen type-1 (COL-1) and hydroxyproline (Hyp.) were detected by ELIZA kits from 3massay, Germany and Cosmo Bio, USA, Catalog No. 20299 and CSB-E08838r, respectively. Total antioxidant capacity (TAC) was determined by BioVision, USA, Catalog No. MBS733414. While the activities of superoxide dismutase (SOD) and catalase (CAT) were detected using Cusabio Biotech, USA, Catalog No. CSB-E08555r and MBS2600683, respectively. Moreover, the oxidative stress marker malondialdehyde (MDA) was estimated by MyBioSource, USA, Catalog No. MBS046858. However, colorimetric methods were used to estimate oxidative stress markers, hydrogen peroxide (H_2_O_2_) by Cell Biolabs, USA, while protein carbonyl (PC) was used by Cayman, USA according to the enclosed methods of Votyakova and Reynolds^28^ and Zusterzeel et al.^29^, respectively.

### Histopathological investigations

#### Hematoxylin and eosin (H&E) sections

For the overall evaluation of lung structural changes by light microscopy, H&E staining was performed. A part of the right lung of the control and different rat groups after necropsy was fixed in 10% formol saline, embedded in paraffin, sectioned at 5 µm thickness, and stained with H&E^30^.

#### Immunohistochemical (IHC) staining

For immuno-labeling, 5 μm sections of each group were loaded on positively charged slides. Monoclonal anti-rabbit primary antibody was applied for detection of Bax (1:75), and Bcl2 (1:50) (Santa Cruz, USA). Localization of bound primary antibodies was conducted after incubation with anti-rabbit secondary antibody for 1 h. Afterwards, development was conducted using Diaminobenzidine (DAB) stain according to manufacturer's protocol (Vector Lab, Tucson, AZ). Three different sections of each group for each primary antibody were examined under bright field Olympus microscope C31 equipped with amscope mu 1000 camera.

#### Quantitative morphometric measurements of Bax and Bcl2

The percent of area appeared with Bax and Bcl2 reactions in lung sections stained by immunostaining reaction was illustrated in each group at X100 magnification by BLeica Quin 500^ image analyzer computer system (Leica image system Ltd.; Cambridge, England).

#### Ultrastructural examination

Transmission electron microscopy (TEM) was performed on lung specimens of different groups fixed in buffered 2.5% glutaraldehyde (pH 7.4) followed by buffered 1% osmium tetraoxide at 4℃. Samples were dehydrated in an ascending series of ethanol, cleared in acetone, and embedded in epoxy resin^31^. Ultrathin sections were cut with LKB Ultratome IV, mounted on grids, stained with uranyl acetate and lead citrate, and examined on a Joel 100CX1 transmission electron microscope (Mansoura University, Egypt).

### Statistical analysis

The obtained data were analyzed using the GraphPad Prism software program (v 5.04 GraphPad Software Inc., La Jolla, CA) using one-way ANOVA followed by Tukey’s test, where statistically significant data were considered at *p* < 0.05. All results were recorded as the mean ± SD.

### Ethical approval

This study was performed in line with the principles of the Declaration of Helsinki. Approval was granted by the Ethics Committee of the Faculty of Science, Mansoura University (No. Sci-Z-P-2021–37).

## Results

### Lung biochemical measurements

**Figure 1:**
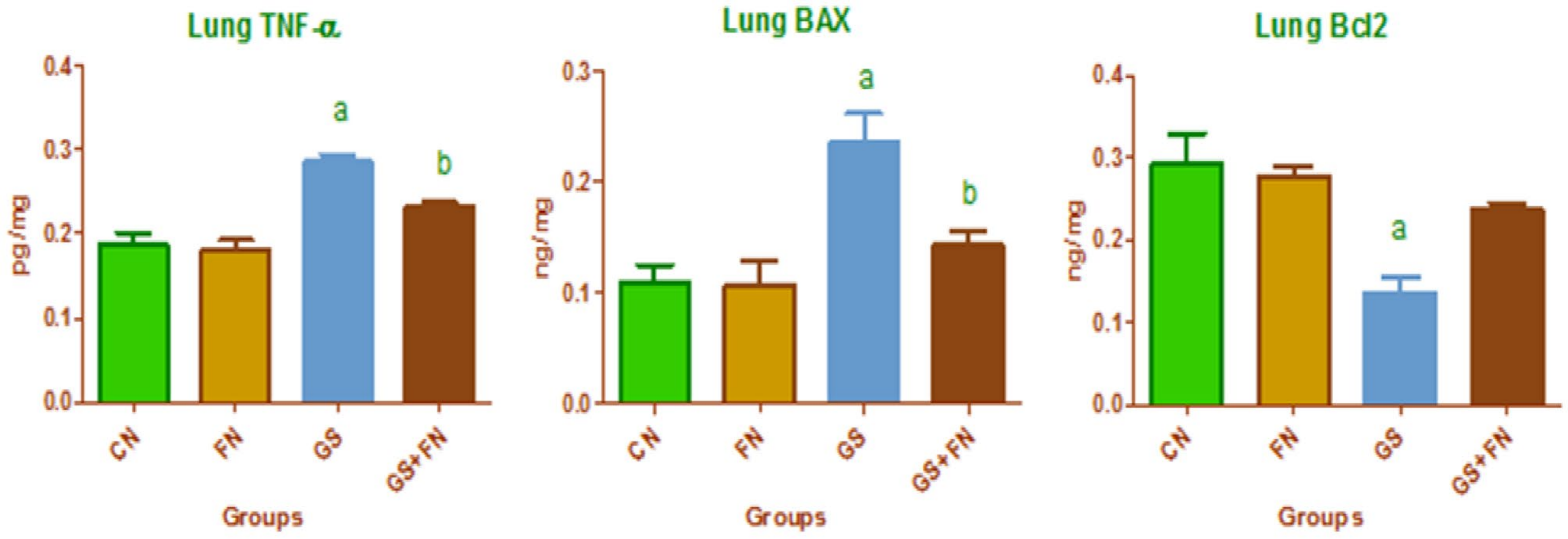
Lung proinflammatory (TNF-α), proapoptotic (Bax), and antiapoptotic (Bcl2) markers in the investigated groups, *CN* control, *FN* fenugreek diet, *GS* gasoline. ^a,b^Superscripts refer to significant changes (*p* < 0.05). a: All groups compared to the CN group, b: GS + FN group compared to the GS group.

**Figure 2:**
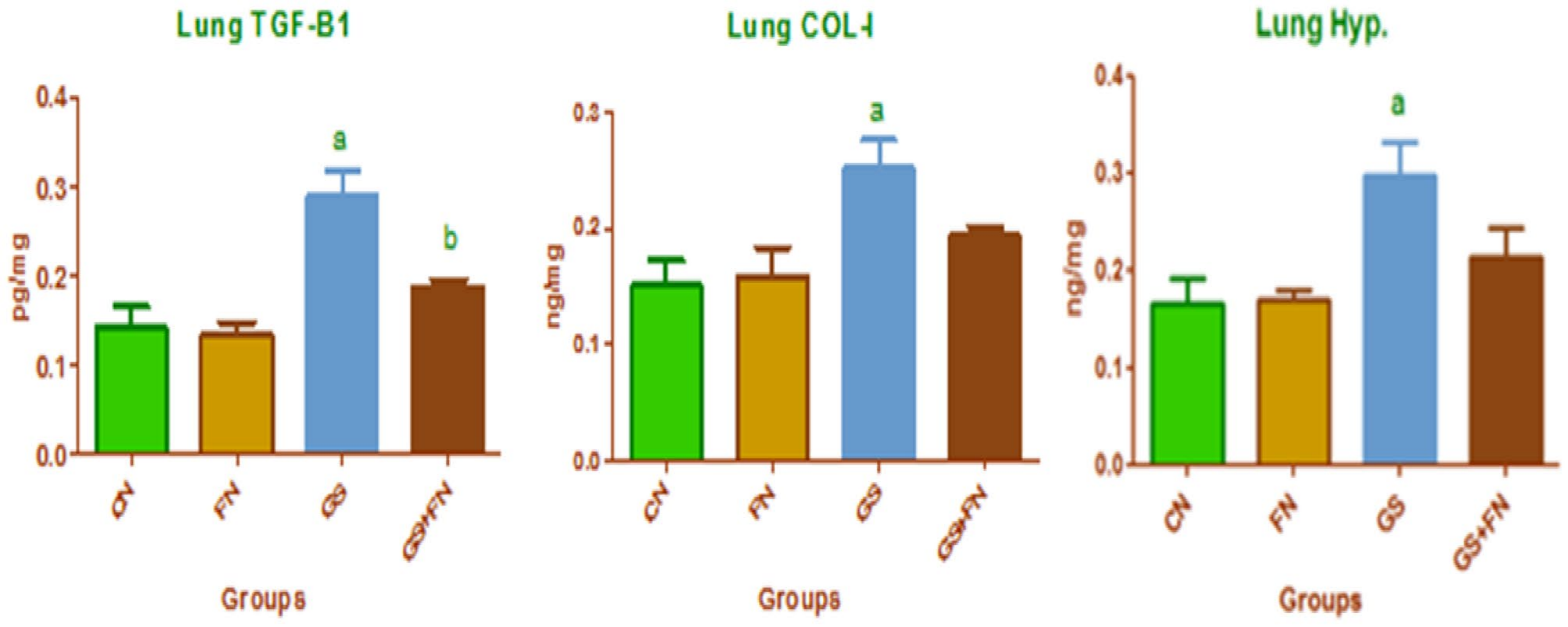
Lung fibrotic markers in the investigated groups, *CN* control, *FN* fenugreek diet, *GS* gasoline. ^a,b^Superscripts refer to significant changes (*p* < 0.05). a: All groups compared to CN group. b: GS + FN group compared to GS group.

The present study revealed a significant increase in the lung proinflammatory marker TNF-α (0.286 ± 0.015 compared to 0.187 ± 0.026 pg/mg) and the proapoptotic protein level of Bax (0.235 ± 0.047 compared to 0.109 ± 0.027 ng/mg) with a marked decrease in the antiapoptotic protein Bcl2 (0.136 ± 0.033 vs 0.291 ± 0.067 ng/mg) in the lung tissue of rats exposed to gasoline fumes compared to the control group (Fig. 1). Moreover, gasoline-exposed rats exhibited significant increases in lung fibrotic markers (TGF-β1, COL-1, and Hyp.) if compared to the control group (0.289 ± 0.051 vs 0.143 ± 0.041 pg/mg, 0.252 ± 0.042 vs 0.150 ± 0.038 ng/mg, 0.298 ± 0.058 vs 0.166 ± 0.047 ng/mg), respectively as seen in Fig. 2. Oxidative stress markers (H_2_O_2_, MDA, and PC) showed significant increases (0.247 ± 0.050 vs 0.126 ± 0.033 mu/mg, 0.305 ± 0.017 vs 0.226 ± 0.017 nmol/mg, 0.281 ± 0.026 vs 0.137 ± 0.041 nmol/mg), respectively. This was accompanied by remarkable decreases in TAC (0.143 ± 0.026 vs 0.217 ± 0.017 ng/mg) and antioxidants (SOD, CAT), (0.091 ± 0.029 vs 0.183 ± 0.030 U/mg, 0.098 ± 0.027 vs 0.189 ± 0.015 ng/mg), respectively in the lungs of gasoline-exposed rats compared to control rats (Table 1). All these findings were markedly improved through the administration of a fenugreek diet with gasoline exposure compared to gasoline-exposed rats, but changes in Bcl2, COL-1, and Hyp. were nonsignificant statistically. Moreover, no obvious differences were detected between the unexposed fenugreek and control groups.

**Table 1:** Oxidative stress markers and antioxidants in the lung of the investigated groups.

Parameters	Groups			
	CN	FN	GS	GS + FN
H_2_O_2_ (mu/mg)	0.126 ± 0.033	0.113 ± 0.025	0.247^a^ ± 0.050	0.147^b^ ± 0.027
MDA (nmol/mg)	0.226 ± 0.017	0.178 ± 0.028	0.305^a^ ± 0.017	0.233 ^b^ ± 0.022
PC (nmol/mg)	0.137 ± 0.041	0.168 ± 0.030	0.281^a^ ± 0.026	0.197 ^b^ ± 0.008
TAC (ng/mg)	0.217 ± 0.017	0.212 ± 0.005	0.143^a^ ± 0.026	0.209 ^b^ ± 0.016
SOD (U/mg)	0.183 ± 0.030	0.187 ± 0.031	0.091^a^ ± 0.029	0.168 ^b^ ± 0.019
CAT (ng/mg)	0.189 ± 0.015	0.196 ± 0.022	0.098^a^ ± 0.027	0.177 ^b^ ± 0.037

Data are presented as means ± SD.

*CN* control, *FN* Fenugreek diet, *GS* gasoline.

^a,b^Superscripts refer to significant changes (*p* < 0.05).

^a^All groups compared to the CN group.

^b^GS + FN group compared to the GS group.

### Hematological parameters

The results also showed a significant reduction in WBC and RBC counts, Hb content, HCT%, MCH, MCV and PLT count (7.30 ± 0.89 vs 12.1 ± 2.00 10^3^/µL, 5.36 ± 1.12 vs 7.73 ± 0.34 10^6^/µL, 12.0 ± 0.82 vs 15.0 ± 0.27 g/dl, 32.0 ± 2.37 vs 43.0 ± 0.96%, 17.9 ± 0.31 vs 20.3 ± 0.30 pg, 48.7 ± 0.65 vs 57.2 ± 1.16 fl, and 529 ± 62.6 vs 754 ± 11.1 10^3^/µL), respectively along with a significant increase in RDW% (21.4 ± 1.47 vs 16.8 ± 0.62%) in the gasoline-exposed rats compared to the control group. These alterations tended to be ameliorated with fenugreek supplementation during gasoline exposure, where a significant increase in RBCs count, Hb content, HCT%, MCV, and PLT count along with a significant decrease in RDW% were observed compared to the gasoline-exposed group. At the same time, no significant changes were observed between the administered fenugreek (FN) group and the control (CN) group, referring to the safe use of this plant with no toxic effects (Table 2).

**Table 2:** Hematological parameters in the investigated groups.

Parameters	Groups			
	CN	FN	GS	GS + FN
WBCs (10^3^/µL)	12.1 ± 2.00	12.7 ± 1.39	7.30^a^ ± 0.89	11.2^b^ ± 1.19
RBCs (10^6^/µL)	7.73 ± 0.34	7.93 ± 0.20	5.36^a^ ± 1.12	7.15^b^ ± 0.64
Hb (g/dl)	15.0 ± 0.27	15.1 ± 0.15	12.0^a^ ± 0.82	13.4^ab^ ± 0.47
HCT (%)	43.0 ± 0.96	43.8 ± 0.51	32.0^a^ ± 2.37	38.1^ab^ ± 2.48
MCH (pg)	20.3 ± 0.30	20.2 ± 0.27	17.9^a^ ± 0.31	19.3 ± 1.01
MCV (fl)	57.2 ± 1.16	58.9 ± 2.15	48.7^a^ ± 0.65	55.4^b^ ± 2.48
RDW (%)	16.8 ± 0.62	16.5 ± 0.32	21.4^a^ ± 1.47	18.5^b^ ± 0.50
PLTs (10^3^/µL)	754 ± 11.1	779 ± 38.2	529^a^ ± 62.6	678^b^ ± 19.1

Data are presented as means ± SD.

*CN* control, *FN* Fenugreek diet, *GS* gasoline.

^a,b^Superscripts refer to significant changes (*p* < 0.05).

^a^All groups compared to the CN group.

^b^GS + FN group compared to the GS group.

### Histopathological changes

#### H&E sections

**Figure 3:**
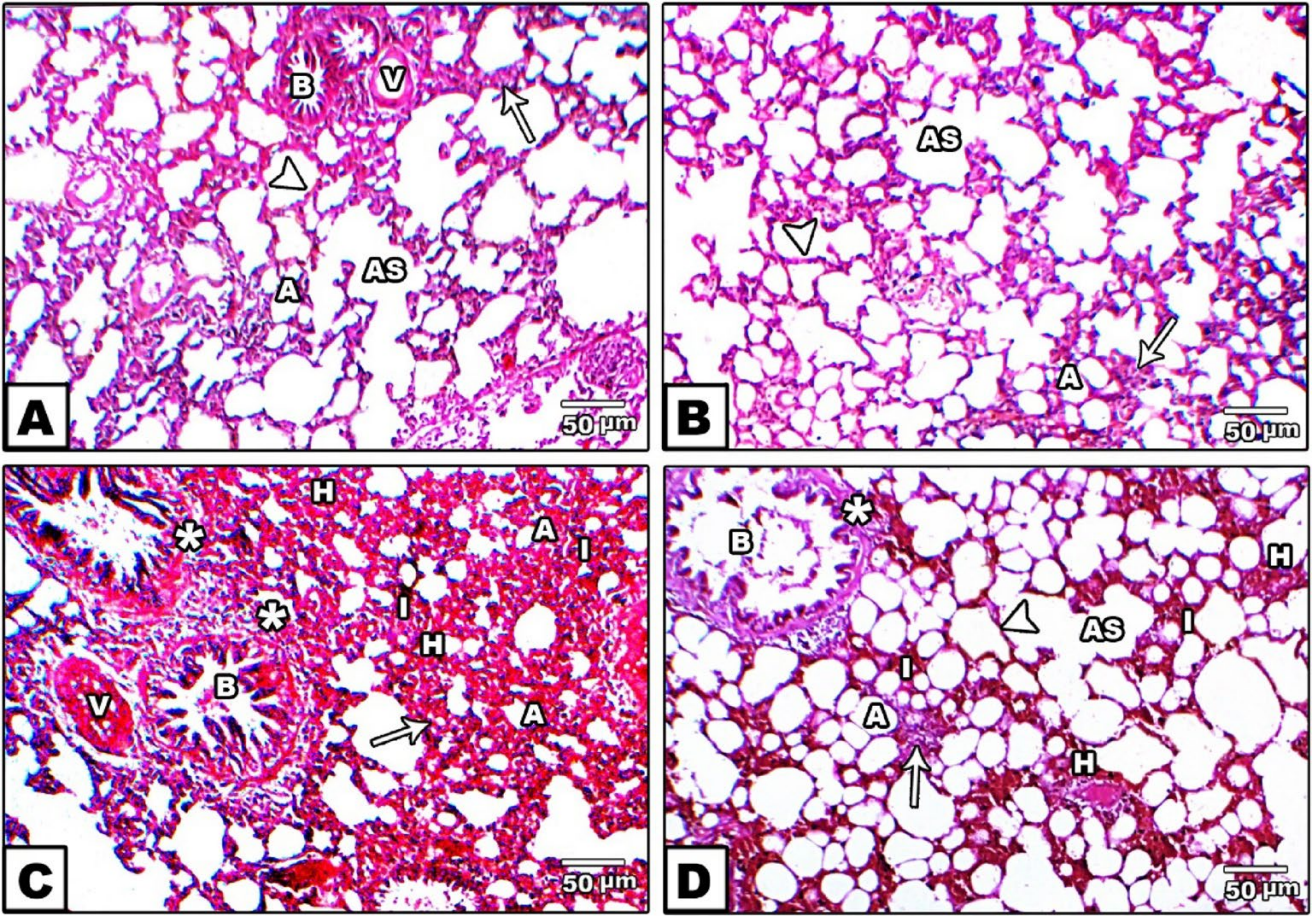
Photomicrographs of H&E stained lungs in the control (**A)** and fenugreek (**B**) groups showing normal lung structures of alveolar sacs (AS), alveoli (A), thin (arrowhead) and thick (arrow) interalveolar septa, in addition to normal lung structures of bronchioles (B) and blood vessels (v) appeared in control group. Gasoline-exposed group (**C**) showing bronchioles (B) with thickening (hypertrophy) of the muscular layer of the bronchial wall (star), congested blood vessels (V), marked proliferation of hemorrhage (H) in interstitial tissue (I) that forming thickening in interalveolar septa (arrow) and collapsed alveoli (A). The gasoline + fenugreek seed powder group (**D**) showed a decrease in hyperplasia of bronchioles (star), reduced proliferation of hemorrhage (H) in interstitial tissue (I), appearance of alveolar sacs (AS), and alveoli (A) with thin (arrowhead) and thick (arrow) alveolar septa compared to the gasoline group.

Lung sections of different studied groups stained by H&E are represented in Fig. 3 and show control (A) and fenugreek (B) groups with normal lung structures of alveolar sacs and alveoli with thin and thick interalveolar septa. In addition, control lung group showed normal lung bronchioles and blood vessels. However, the gasoline-exposed group (C) exhibited thickening (hypertrophy) of the muscular layer of the bronchial wall, congested blood vessels, and marked proliferation of interstitial tissue showing hemorrhage and forming thickened interalveolar septa with a decreased number of alveoli that were collapsed. However, the lungs of rats in the gasoline + fenugreek group (D) showed diminished hypertrophy of bronchioles, collapsed alveoli with thin and thick alveolar septa, and decreased hemorrhage, indicating lung amelioration with fenugreek seed supplementation during gasoline exposure.

#### Immunohistochemical (IHC) staining

**Figure 4:**
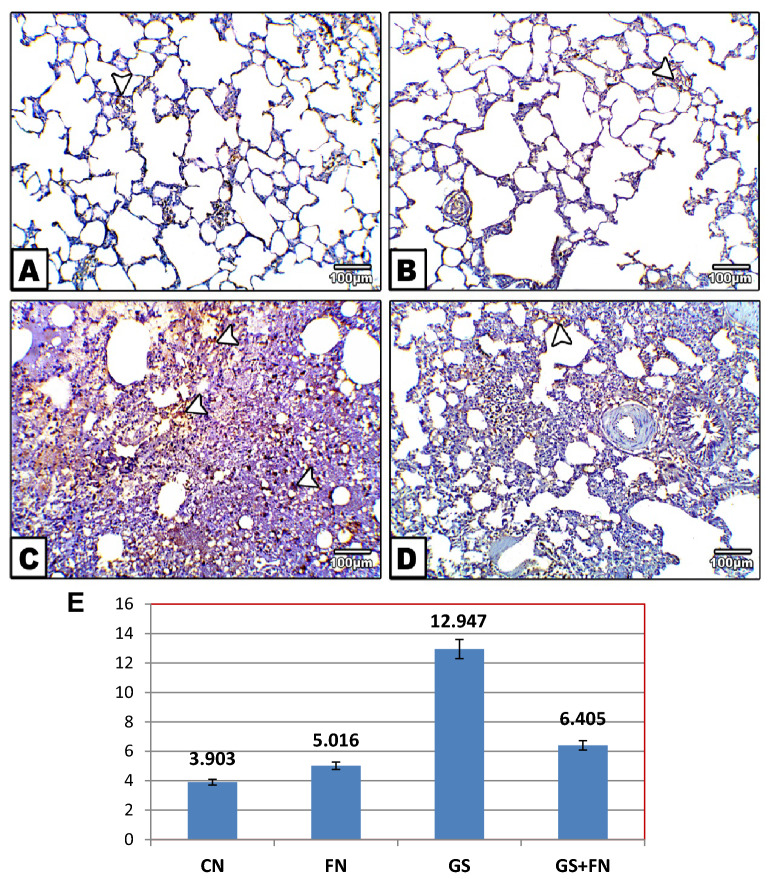
Photomicrographs of histological sections of lung immunohistochemically labeled by Bax primary antibody. Unexposed control and fenugreek groups (**A**,**B**) showing very little immunohistochemical reaction of Bax. The gasoline-exposed group (**C**) showed an increase in the dark brown color of the immunohistochemical reaction of Bax in inflamed lung cells. Gasoline-exposed group-administered fenugreek seeds (**D**) showed a decrease in the immunohistochemical reaction of Bax. Arrowhead pointed to the increase in the immunohistochemical reaction of Bax. (**E**) Histogram showing the percentage of immunohistochemical reaction of Bax in the lung of rat groups using image analysis.

The BAX immune reaction was less detected in the alveolar tissue of both the unexposed control and fenugreek groups (Fig. 4A,B). However, immunohistochemical staining showed a marked increase in the content of Bax in the alveolar tissue, which appeared dark brown in color in the gasoline-exposed group, indicating increased cell apoptosis (Fig. 4C) compared to the control. Furthermore, the reaction of the apoptotic immune marker (Bax) appeared to be mild with the administration of fenugreek seeds during exposure to gasoline when compared to the gasoline group (Fig. 4D).

**Figure 5:**
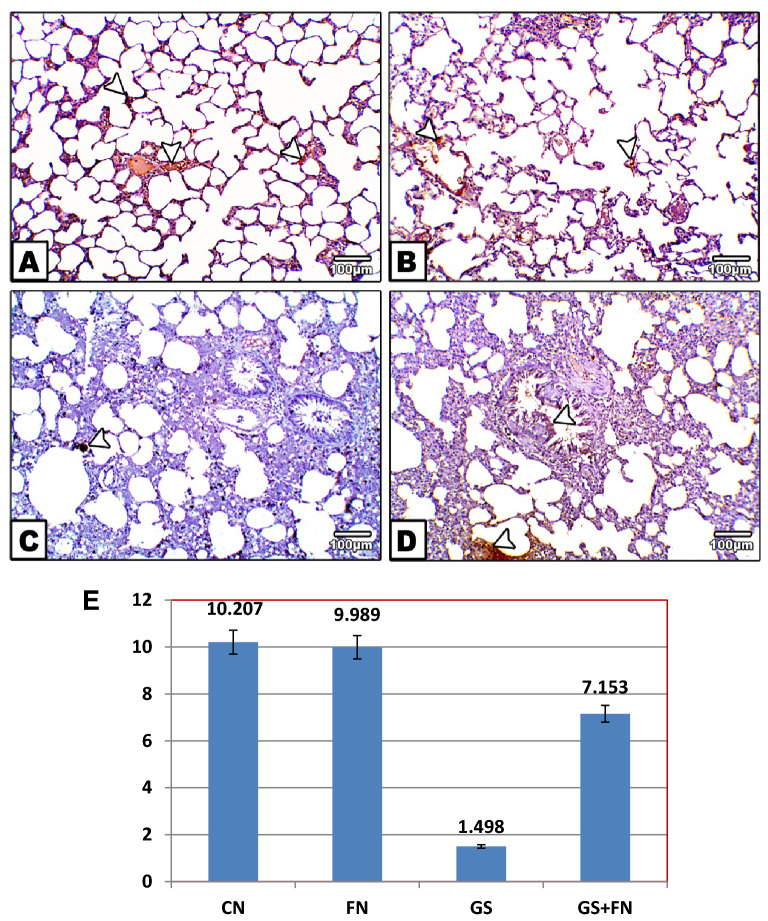
Photomicrographs of histological sections of lung immunohistochemically labeled with Bcl2 primary antibody. Unexposed control and fenugreek groups (**A**,**B**) showed alveolar tissue rich in the dark brown immunohistochemical reaction of Bcl2. The gasoline-exposed group (**C**) showed no immunohistochemical reaction of Bcl2 in inflamed lung cells. The gasoline-exposed group-administered fenugreek seeds (**D**) showed a mild increase in the immunohistochemical reaction of Bcl2 in lung cells. Arrowhead pointed to the increase in the immunohistochemical reaction of Bcl2. (**E**) Histogram showing the percentage of immunohistochemical reaction of Bcl2 in the lung of rat groups using image analysis.

On the other hand, Bcl2 increased the immune reaction in normal alveolar cells of the control and fenugreek groups, where a dark brown color was deposited (Fig. 5A,B). However, no obvious color was observed in the gasoline-exposed group, indicating the cell tendency for apoptosis (Fig. 5C), but with fenugreek administration during gasoline exposure, the Bcl2 reaction was moderately observed in alveolar cells, indicating the role of fenugreek seeds in counteracting the proapoptotic effect of gasoline (Fig. 5D).

#### Quantitative morphometric measurements of Bax and Bcl2

Morphometric results for area percent of Bax protein expression in lung sections showed high percent area of brown color with exposure to gasoline fumes compared to the control. This percent was decreased with fenugreek administration during gasoline exposure to values near the control group, as shown in Fig. 4E. However, Bcl2 protein expression exhibited low percent area with exposure to gasoline fumes, that was increased with fenugreek seed supplementation compared to gasoline-exposed group (Fig. 5E).

#### Ultrastructural changes

**Figure 6:**
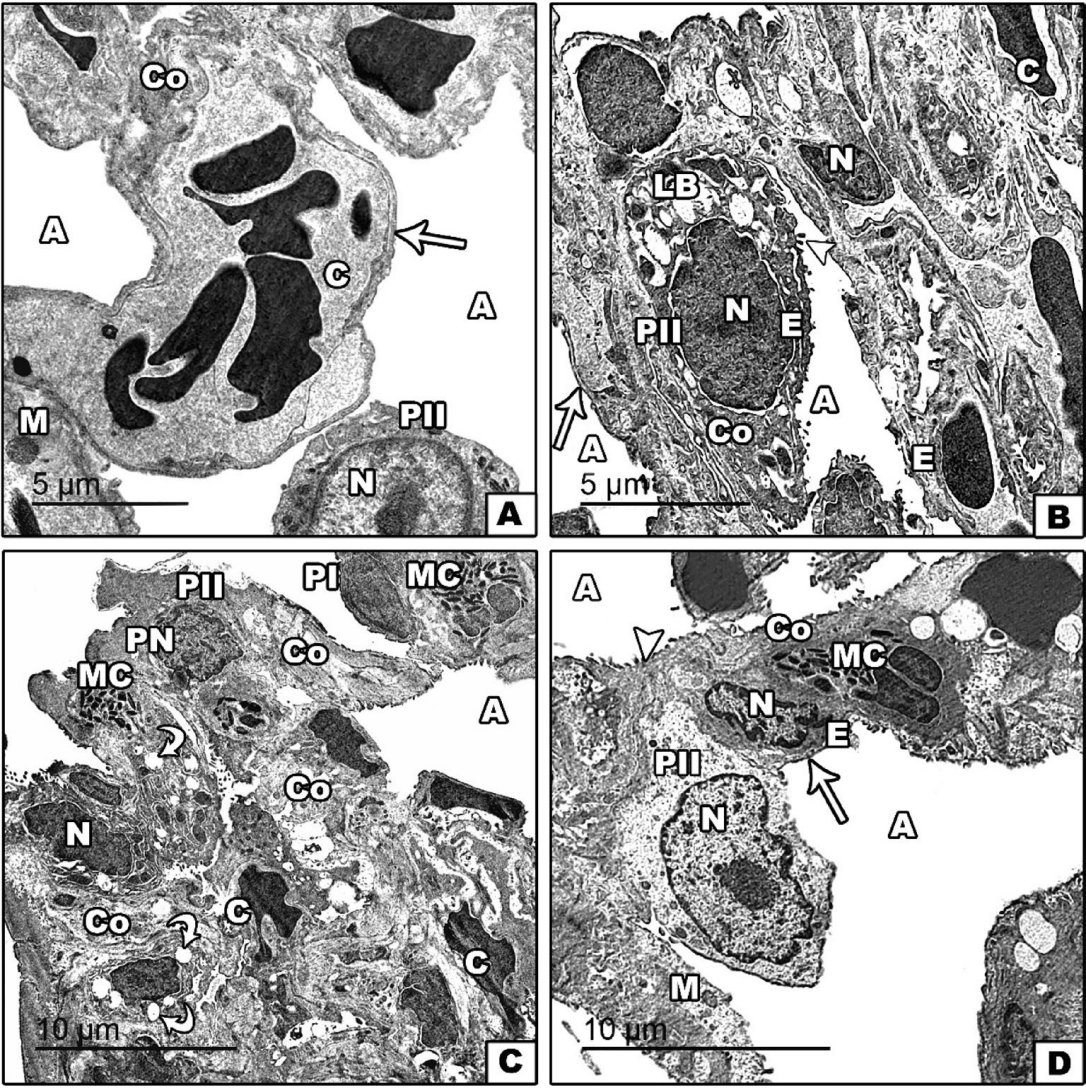
Transmission electron micrographs of the lung tissue of unexposed control and fenugreek groups (**A** and/or **B**) showing normal alveoli (A), with thin alveolar wall (arrow), normal pneumocytes II (PII), endothelial cells (E), nucleus (N), microvilli (arrowhead), mitochondria (M), and supplied with blood capillaries (C), some collagen fibers (Co) and lobular bodies (LB). The gasoline-exposed group (**C**) showed pneumocyte I (PI) and pneumocyte II (PII) with pyknotic nuclei (PN), the appearance of dense collagen fibers (Co), increasing mast cells (MC), cytoplasmic vacuoles (curved arrow), and congested blood capillaries (C). Fenugreek administration with gasoline exposure (**D**) showed relative improvement in alveoli (A), with thin alveolar wall (arrow), endothelial cells (E), microvilli (arrowhead), pneumocyte II (PII) with nucleus (N), mitochondria (M), and decreased numbers of mast cells (MC) and collagen fibers (Co).

At the ultrastructural level, TEM in the lungs of both the control and fenugreek groups showed normal alveoli with thin alveolar wall and microvilli. Alveoli composed of normal pneumocytes II with large nucleus occupied almost the cell body. Rough endoplasmic reticulum and mitochondria were observed. Moreover, some cells have several vacuoles, and electron-dense inclusions. Additionally, the lining wall is provided with numerous blood capillaries and some collagen fibers (Fig. 6A,B). However, the lungs of the gasoline-exposed group showed several pathological changes represented by pyknosis of compact dense chromatin materials of type II pneumocytes and pneumocyte I. The cytoplasm contained rough endoplasmic reticulum and cytoplasmic vacuoles. Moreover, a considerable increase in the number of mast cells in inflamed alveolar tissue was also observed. In addition, large masses of collagen fibers were noticed in interstitial tissue of the alveolar wall, in addition to the appearance of damaged blood capillaries (Fig. 6C). On the other hand, supplementation of fenugreek seeds with exposure to gasoline fumes improved pathological signs, including the appearance of normal structures of pneumocyte II with nuclei and cytoplasmic granules. Microvilli and mitochondria were observed, In addition to a decrease in deposited collagen fibers and mast cells (Fig. 6D).

## Discussion

Misuse or occupational exposure to leaded gasoline fumes can adversely affect various body organs and systems^24,32^. However, the respiratory tract seems to be more vulnerable to gasoline toxicity due to its large surface area, which makes gasoline fumes readily absorbed by the lung, causing lung toxicity^33^. The present study mimicked occupational exposure to leaded gasoline fumes for 6 h/day, 6 days/week for 10 weeks. The obtained results showed that rats exposed to gasoline fumes exhibited a significant increase in TNF-α, an inflammatory marker, compared to the control group. This inflammatory cytokine was reported to be released from mast cells recruited to the inflammatory site^34^. Several studies have supported this, as under normal physiological conditions, no mast cells can be detected in the lung, while in the case of inflammation, they can be observed^34,35^. This was broadly consistent with the present study through lung TEM sections, where mast cells were observed in the gasoline-exposed group compared to the control group. In this regard, WBCs count in circulating blood is one of the systemic inflammatory responses in the body^36^. Although the evidence of leukocytosis in the literature was inconsistent, the present study showed a significant decrease in blood WBCs count with exposure to gasoline fumes compared to the control. Jabbar and Ali^11^ attributed this to chronic exposure to petroleum products that could reduce leucocyte number in a way affecting the immune process making the body more vulnerable to pathological pathways. Moreover, the present study dealt with leaded gasoline, where lead was reported to affect the immune response either in humans^11^ or animals^9^. Amor-Carro et al.^15^ explained that lead exposure could stimulate cascade events, including oxidative stress and inflammation, accompanied by reduced immune status. Moreover, the current study exhibited a significant increase in Bax expression and a decrease in Bcl2. This was in accordance with Wang et al.^6^, who found a strong relationship between the body inflammatory response and apoptotic reaction. Wang et al.^6^ explained that increasing the degree of inflammation may lead to activation of pathological apoptosis signaling pathways, increased expression of Bax, as proapoptotic protein, and decreased expression of Bcl2, an antiapoptotic protein. Immunohistochemical investigations and morphometric analysis in the current study also supported this result, which may reflect the death of the alveolar epithelium in the gasoline-exposed group compared to the control. Therefore, the inflammatory response is a highly regulated process through which the balance between cell survival and apoptosis occurs to drive and resolve inflammation. On the other hand, the obtained data showed a significant increase in TGF-β1, COL-1, and Hyp, fibrotic markers, in the lungs of gasoline-exposed rats compared to control rats. Shen et al.^37^ attributed this to phagocytes that try to clear apoptotic cells and contribute to resolution by generating TGF-β1. However, the increased production of TGF-β1 stimulates the migration and proliferation of fibroblasts to deposit extracellular matrix, particularly COL-I as the most abundant collagen of the body, to initiate the repair process^38^. In addition, Hyp a major component of collagen, was significantly increased in the gasoline-exposed group, reflecting a case of pulmonary fibrosis^39^.

Furthermore, Amor-Carro et al.^15^ attributed cell death to the increased formation of ROS, which leads to oxidative damage of mitochondrial DNA. Su et al.^40^ recommended that lipid peroxidation may serve as a common mediator of apoptosis in response to toxicants and pathological conditions. El-Sayed^41^ added that highly oxidized lipids from gasoline exposure could attack nearby proteins forming more protein carbonyls that are accompanied by several inflammatory reactions. The present study was in accordance with these findings, where significantly increased levels of oxidative stress markers (H_2_O_2_, MDA and PC) in the lungs of rats exposed to gasoline fumes compared to the control group were observed. Elevated levels of H_2_O_2_ reflect increased formation of ROS, which could attack the lipoprotein membrane of the lung, causing oxidation of both lipids and proteins, yielding MDA and PC, respectively. Moreover, increased production of H_2_O_2_ could inhibit parenchymal cells of the lung to produce antioxidants, which may explain the decreased levels of TAC, SOD and CAT causing an imbalance in the lung oxidant/antioxidant levels^2^. In turn, this could result in loss of cell and tissue integrity, as seen in the current study, through histopathological changes in the lungs of the exposed group compared to the control group. This was in accordance with Roda et al.^42^, who attributed hemorrhage to the leakage of fluid into the extravascular space because of lipid and protein oxidation. The authors added that exposure to ROS harms type II pneumocytes, resulting in alveolar collapse, and interstitial inflammation, as seen in H&E and TEM lung sections in the present study.

On the other hand, blood is the most important body fluid that controls respiration^43^ and primarily provides useful information on general health after exposure to extrinsic damage^11^. The present study also indicated a significant reduction in RBCs count, Hb content, HCT%, MCV, and MCH in gasoline-exposed rats compared to the control. These results agreed with the data of Teklu et al.^44^, who attributed this to the cytotoxic effects of the benzene constituent of gasoline, which depresses hematopoiesis in bone marrow, causing aplastic anemia. Moreover, naphthalene, as one of the gasoline constituents, is also known to affect the red cell membranes, leading to hemoglobin denaturation and hemolytic anemia^45^. Furthermore, Nathan et al.^46^ reported that low Hb content accompanied by elevated levels of RDW% (measures the variation in RBC size) was previously mentioned to be related to pulmonary disorders caused by an underlying state of pulmonary inflammation accompanied by changes in erythropoiesis. This agreed with the present study supporting the obtained lung inflammatory status. Moreover, the decreased PLT count (thrombocytopenia) in gasoline-exposed rats may be related to the formation of lipid peroxides within platelet membranes, thus provoking platelet lysis and decreasing the platelet count. This may participate in pulmonary hemorrhage, as seen in intraalveolar and interstitial areas of the lungs of the gasoline-exposed group compared to the control (H&E sections), suggesting disrupted gas exchange and lung function^42^.

Nutritive natural plants have been reported to play an essential role in alleviating several health problems^16^. Fenugreek seeds possess several phytochemicals, including vitamins, flavonoids, alkaloids, terpenoids, carotenoids, coumarins, curcumins, lignin, and saponins^18^. Although there is no direct study between the addition of fenugreek seed powder to food and the lung response during gasoline exposure, several investigators have demonstrated the anti-inflammatory and antioxidant potential of fenugreek seeds in experimental animals^19,24,47^. Abdrabouh^24^ attributed this to the high levels of phenolics and tannins, represented in gallic acid, in addition to quercetin and saponins, as well as lower values of radical scavenging activity (IC50%). Bafadam et al.^22^ and Yusharyahya^47^ reported that the interesting activities of fenugreek seeds are related to higher amounts of trigonelline and diosgenin steroids as well as the alkaloid and flavonoid contents. However, Tewari et al.^17^ attributed the high antioxidant potential of fenugreek seeds to the reduction of ROS, which leads to a feedback regulation loop suppressing the levels of oxidative stress markers. This may explain the obtained significant decrease in oxidative stress markers (H_2_O_2_, MDA and PC) with fenugreek administration during gasoline exposure along with increased TAC, SOD and CAT as antioxidants. Indeed, the amelioration of oxidant/antioxidant balance may participate in the inflammatory status induced by gasoline inhalation. This was clear in the pronounced decrease in TNF-α and mast cells in TEM sections compared to the gasoline-exposed group. Bafadam et al.^22^ explained that fenugreek seeds could inhibit the production of phorbol-12-myristat-13-acetate induced inflammatory cytokines such as TNF-α. However, Emtiazy et al.^48^ attributed the anti-inflammatory effect of fenugreek seeds to flavonoids, especially quercetin, which inhibits the activation of mast cells. Another explanation was provided by Durga et al.^49^, where quercetin ligands interact with the protein constituent of cytokines, forming hydrogen bonds and then suppressing their over release. In turn, apoptotic and antiapoptotic proteins were significantly ameliorated compared to those in the gasoline-exposed group, as shown by biochemical and immunohistochemical analyses supported by image analysis. The percentage of Bax decreased and Bcl2 increased, reflecting the antioxidant, anti-inflammatory and antiapoptotic activities of fenugreek seeds. This may explain the consequent significant decrease in TGF-β1 along with COL-I and Hyp in the gasoline group administered fenugreek seeds compared to the gasoline group.

These observations were also accompanied by a significant increase in WBCs count, reflecting the potential role of fenugreek seeds in increasing the immune response. Moreover, fenugreek seeds are rich in amino acids (lysine and threonine), minerals (iron and copper), and vitamins (folate and ascorbic), which are all essential components of hemoglobin synthesis^50^. This may explain the observed alleviation of blood Hb and other blood indices with fenugreek administration compared to the gasoline group. As a result, histopathological changes in the lung showed pronounced improvement in most pulmonary architecture in the exposed group administered fenugreek seeds compared to the gasoline group. Yao et al.^51^ attributed this to the high antioxidant activity of fenugreek seeds, which could restore oxidant/antioxidant balance.

In conclusion, exposure to gasoline fumes is intimately related to lung inflammatory and proapoptotic disorders resulting from released ROS and helps release fibrotic factors that affect the lung architecture. However, supplementation with a fenugreek seed diet during gasoline exposure helped to alleviate all biochemical and histological alterations in alveolar tissue. Thus, fenugreek seed powder is a powerful plant that is recommended to add to food, especially for those exposed to gasoline fumes.

## Data Availability

All data generated or analyzed during this study are included in this published article.
